# The Relationship between Endogenous Androgens and Body Fat Distribution in Early and Late Postmenopausal Women

**DOI:** 10.1371/journal.pone.0058448

**Published:** 2013-03-04

**Authors:** Yuankui Cao, Shaofen Zhang, Shien Zou, Xian Xia

**Affiliations:** Department of Gynecology, Obstetrics and Gynecology Hospital of Fudan University, Shanghai, China; Ecole Normale Supérieure de Lyon, France

## Abstract

**Objectives:**

To investigate the relationship between endogenous androgens and body fat distribution in early and late postmenopausal women.

**Materials and Methods:**

We enrolled postmenopausal women consisting of an early group (≤5 years since menopause, n = 105) and a late group (≥10 years since menopause, n = 107). Each group was subdivided into normal weight (BMI <24 kg/m^2^) group, overweight and obese (BMI ≥24 kg/m^2^) group. Fasting total testosterone (T), dehydroepiandrosterone-sulfate (DHEA-S) and sex hormone-binding globulin (SHBG) levels were measured. Body fat distribution was evaluated by dual-energy X-ray absorptiometry (DEXA).

**Results:**

Late postmenopausal women had a higher proportion of body fat than early postmenopausal women. The body fat of the overweight and obese women had a greater tendency to accumulate in the abdomen compared with the normal weight women both in early and late postmenopausal groups. The overweight and obese women had a higher free testosterone (FT) than the normal weight women in early postmenopausal women (*P*<0.05). In late postmenopausal women, the overweight and obese women had higher DHEA-S levels than normal weight women (*P*<0.05). No direct relationship was observed between the T levels and body fat distribution both in early and late postmenopausal groups (*P*>0.05).The FT in early postmenopausal women and the DHEA-S levels in late postmenopausal women correlated positively with the trunk/leg fat ratio (T/L) and the proportion of android fat whereas correlated negatively with the proportion of gynoid fat in the partial correlation and multiple linear regression analyses (all *P*<0.05).

**Conclusions:**

Serum T levels do not correlate directly with body fat distribution, the FT in early postmenopausal women and DHEA-S levels in late postmenopausal women correlate positively with abdominal fat accumulation.

## Introduction

Previous researchers have demonstrated that postmenopausal women tend to gain weight and that menopause is associated with a preferential increase in intra-abdominal fat [1], [2], [3]. Body fat may be redistributed and tends to accumulate more in the upper body, especially the abdomen, after menopause [4], [5].It is well known that abdominal obesity significantly increases the risk of developing metabolic syndrome (MS),diabetes and coronary heart disease(CHD) [6], [7],which are detrimental to the health of elderly women.

Both observational and clinical trials suggest that estrogen deficiency is associated with increased adiposity and that a low dose of estrogen therapy before 60 years could decrease abdominal fat accumulation [8], [9]. Sex hormone-binding globulin (SHBG) levels decrease due to the decline of estrogens after menopause, which results in an increase of free testosterone (FT) [10]. Previous studies have suggested that elevated FT levels are associated with an accumulation of abdominal fat in premenopausal women [11], [12], [13], [14]. However, few studies have been performed in postmenopausal women, and the results have not always been consistent. Furthermore, in late postmenopausal women, the majority of testosterone originates from dehydroepiandrosterone(DHEA) or dehydroepiandrosterone-sulfate(DHEA-S)in peripheral intracrine tissues [15]. Both ovaries and adrenals produce DHEA/DHEA-S. The postmenopausal ovary is hormonally active, contributes significantly to the circulating pool of androgens and this contribution appears to persist in women as long as 10 years beyond the menopause [16].However, as a result of ovarian failure, the approximately 20% contribution of the ovary to the total pool of DHEA/DHEA-S doesn’t secret androgens directly [17].Thus in late postmenopausal women, the peripheral synthesis by the conversion of DHEA\DHEA-S originating from the adrenals becomes the main source of androgens [17], [18]. DHEA sulfotransferase catalyzes the transformation of DHEA to DHEA-S and this enzyme is mainly expressed in adrenals [19]. DHEA-S has a longer half-life period and is more stable in the circulation than DHEA. So DHEA-S levels could partly reflect the levels of androgens derived from adrenals in late postmenopausal women. It is unclear whether DHEA-S is associated with body fat distribution.

As ovarian ageing is a long process during natural menopause, the relationship between androgens and body fat distribution may be different in early and late postmenopausal women. In order to highlight this difference, we recruited participants in two groups according to the number of years since menopause. Therefore, the aim of our study was to evaluate the relationship between endogenous androgens and body fat distribution in early and late postmenopausal women.

## Materials and Methods

### Participants

This cross-sectional analysis was conducted on postmenopausal women aged 46 to 85 years who were recruited from the community around the Gynecology & Obstetrics Hospital of Fudan University between 2009 and 2011. The study received ethics approval from the Human Ethics Committee of the Gynecology & Obstetrics Hospital of Fudan University. All people in both groups voluntarily joined this study and signed the informed consents.

Women with a diagnosis of polycystic ovary syndrome, thyroid disease, chronic renal failure, chronic liver disease, cancer, diabetes, hypertension, coronary cardiovascular disease, or obesity due to other known diseases (e.g. cushing syndrome, hypothalamus disease) were excluded from the study. Women who were smokers; alcohol drinkers; or taking contraceptive drugs, hormone therapy medications, or any other drugs (e.g. adrenocortical hormones, yeast, chloropromazine, reserpine, isoniazide) known to interfere with body weight were also excluded. The presence of medical conditions was assessed through self-reporting.

Menopause was defined as natural menopause with amenorrhea for at least 12 months and a follicle-stimulating hormone level>40 IU/L [20], [21]. Women with surgical menopause or premature ovarian failure were excluded.

A total of 212 women consisting of two groups were eligible for this study: early postmenopausal women (≤5years since menopause) and late postmenopausal women (≥10 years since menopause). The early postmenopause is usually defined as≤5 years since the final menstrual period (FMP) [22]. However, the definition of late postmenopause is still controversial: it was thought to be >5 years since the FMP in the STRAW staging system published in 2001 [22], some experts thought it should be ≥10 years since the FMP, while in the STRAW staging system published in 2012 it was revised to >6 years since the FMP [23]. Whether the intermediate postmenopausal women (6–9 years since the FMP) are assigned to early postmenopause or late postmenopause is ambiguous and we excluded these women from this study.

Based on the guidelines defining overweight and obese Chinese adult published in 2002, a body mass index (BMI) ≥24 kg/m^2^ was defined as overweight and obesity [24], [25]. Each menopausal group was subdivided into two groups: normal weight group (BMI<24 kg/m^2^), overweight and obese group (BMI≥24 kg/m^2^).

### Anthropometric Measurements

Weight and height were measured in light clothing without shoes. Body height was measured using a stadiometer, and body weight was measured using a digital electronic scale.BMI was calculated as weight in kilograms divided by the square of height in meters. Waist and hip circumferences were measured using a flexible measuring tape. Waist circumference was measured midway between the xiphoid and the umbilicus during the mid-inspiratory phase [26]. Hip circumference was measured at the widest part of the gluteal region. The waist-to-hip ratio (WHR) was calculated as Waist circumference/Hip circumference.

A body fat composition analysis was performed using dual-energy X-ray absorptiometry (DEXA) (Discovery A, Hologic).The proportion of body fat was calculated as total fat/total mass. The proportion of android fat was defined as (trunk fat +arm fat)/(total body fat). The proportion of gynoid fat was defined as (leg fat)/(total body fat) [27]. A high trunk/leg fat ratio (T/L), a high proportion of android fat, and a low proportion of gynoid fat were used as indications of body fat accumulation in the abdomen.

### Laboratory Analyses

Participants fasted for 12 h and avoided heavy physical activity for 2 h before each examination. Fasting blood samples were collected and extracted by centrifugation at 3000×g for 10 min. Aliquots of serum were immediately stored at −80°C and shipped to the hospital laboratory for long-term freezer storage until analyzed. Serum hormone concentrations were measured from stored samples. This study in which estrogen levels were not measured focused on the relationship between endogenous androgens and body fat distribution. Total testosterone(T), DHEA-S, and SHBG were determined with an immuneanaylzer using chemiluminescence kits (Beckman Coulter, Inc.). To assess the influence of free testosterone on body fat distribution, we calculated FT according to the Vermeulen method [28]. Including approximately 5% blind quality control samples in each batch of samples analyzed was performed to monitor assay variability. The quality control serum was obtained from a large pool that was aliquoted into storage vials and labeled identical to participant samples. The overall coefficients of variation were 3.93% for total T, 6.4% for DHEA-S, and 4.5% for SHBG.

### Statistical Analyses

For the descriptive statistics, the means and standard deviations of the continuous variables were used to describe the study groups. Student’s *t* tests or Wilcoxon rank-sum tests were performed to compare the differences between two groups (early postmenopausal group vs. late postmenopausal group, normal weight group vs. overweight and obese group in early postmenopausal women, normal weight group vs. overweight and obese group in late postmenopausal women).Before the statistical analysis, the normal distributions and homogeneities of the variances were tested. Any parameters that did not satisfy these tests were analyzed using non-parametric rank-sum tests. The correlations between androgens and body fat distribution were evaluated using Pearson’s correlation and multiple linear regression analyses. Partial correlations were adjusted for age and BMI. The model^1^ used in multiple linear regression analysis included T, DHEA-S, SHBG, BMI and age. FT was added to model^1a^. DHEA-S, FT, BMI and age were included in model^2^. SHBG was added to model^2a^.All statistical analyses were performed using SPSS 17.0 software. The statistical significance level was set at *P*<0.05.

## Results

The patient characteristics (mean±SD) for each study group are shown in Table 1. Late postmenopausal women had a higher proportion of body fat compared with early postmenopausal women (34.86±4.29% vs. 33.05±3.50%; *P*<0.01).No significant differences were observed in the body fat distribution, BMI, or WHR between the groups (all *P*>0.05). Early postmenopausal women had higher DHEA-S (112.06±53.19 vs. 92.88±49.28 µg/dL; *P*<0.05) levels than late postmenopausal women. Early and late postmenopausal women had similar T,FT and SHBG levels.

**Table 1:** Comparisons of participants’ characteristics between two groups.

	early postmenopausal women	late postmenopausal women	*p*
	N = 105	N = 107	
Ages(years)	54.58±2.98	69.41±7.28	<0.01
Years since menopause	3.50±1.48	19.35±7.50	<0.01
Weight(kg)	60.47±7.06	58.58±10.07	NS
Height(m)	1.59±0.05	1.58±0.05	NS
BMI(kg/m^2^)	24.06±2.72	23.52±3.28	NS
Waist circumferences(cm)	78.78±7.71	77.63±10.21	NS
Hip circumferences(cm)	92.96±5.36	92.70±7.88	NS
WHR	0.85±0.05	0.84±0.06	NS
T(ng/mL)	0.26±0.15	0.25±0.15	NS
DHEA-S(µg/dL)	112.06±53.19	92.88±49.28	<0.05
SHBG(nmol/L)	61.79±38.12	64.05±27.47	NS
FT(pmol/L)	11.23±9.93	10.07±7.93	NS
Body fat distribution			
Total mass(kg)	59.14±7.15	57.57±10.21	NS
Trunk fat(kg)	10.21±2.46	10.58±3.23	NS
Left arm fat(kg)	1.23±0.27	1.36±0.50	NS
Right arm fat(kg)	1.28±0.30	1.42±0.56	NS
Left leg fat(kg)	3.03±0.70	3.06±0.99	NS
Right leg fat(kg)	3.12±0.74	3.11±1.01	NS
Total fat(kg)	19.68±3.88	20.37±5.96	NS
Proportion of body fat(%)	33.05±3.50	34.86±4.29	<0.01
T/L	1.70±0.40	1.69±0.17	NS
Proportion of android fat	0.64±0.05	0.65±0.04	NS
Proportion of gynoid fat	0.31±0.05	0.30±0.04	NS

Results are expressed as mean±SD. NS, no significant difference; P<0.05, significant difference.

After adjustment for BMI, we found that overweight and obese women had a higher T/L, a higher proportion of android fat, and a lower proportion of gynoid fat than normal weight women both in the early and late postmenopausal groups (all *P*<0.05, Table 2). In the early postmenopausal group, overweight and obese women had higher DHEA-S and T levels than normal weight women, but these differences were not significant. Overweight and obese women had a higher FT (13.85±11.47 vs. 8.49±7.15 pmol/L; *P*<0.05) and lower SHBG levels (52.30±37.32 vs. 71.29±36.93 nmol/L; *P*<0.05) than normal weight women in the early postmenopausal group. In the late postmenopausal group, overweight and obese women had higher DHEA-S (106.10±52.64 vs. 79.65±42.78 µg/dL; *P*<0.05) levels, higher T levels and FT values (not significant), lower SHBG levels (not significant) than normal weight women.

**Table 2:** Body fat distribution and androgens after adjustment for BMI.

	early postmenopausal women		late postmenopausal women			
	BMI<24	BMI≥24	BMI≥24		BMI≥24	
	n = 53	n = 52	n = 53		n = 54	
T/L	1.60±0.35	1.81±0.42*		1.63±0.11		1.74±0.20**
Proportion of android fat	0.63±0.05	0.66±0.04*		0.63±0.04		0.67±0.04**
Proportion of gynoid fat	0.33±0.05	0.30±0.04*		0.32±0.04		0.29±0.05**
DHEA-S (µg/dL)	101.50±50.87	122.62±53.94		79.65±42.78		106.10±52.64**
T (ng/mL)	0.22±0.13	0.29±0.15		0.22±0.13		0.28±0.16
SHBG (nmol/L)	71.29±36.93	52.30±37.32*		70.93±27.03		57.16±26.70
FT(pmol/L)	8.49±7.15	13.85±11.47*		7.08±5.65		13.16±8.82

* p<0.05, overweight women vs. normal weight women in the early postmenopausal group.

** p<0.05, overweight women vs. normal weight women in the late postmenopausal group.

The Pearson’s correlation analyses indicated that there were no direct relationships between the T levels and body fat distribution both in early postmenopausal women and late postmenopausal women (all *P*>0.05, Table 3).

**Table 3:** The relationship between androgens and body fat distribution in early and late postmenopausal groups after adjustment for age and BMI.

	T/L		Proportion of android fat		Proportion of gynoid fat		
	r-value	p-value	r-value	p-value	r-value	p-value	
early postmenopausal women							
T	0.076	0.474	−0.026	0.805	0.017	0.874	
SHBG	**−0.221**	**0.034**	**−0.279**	**0.007**	**0.270**	**0.009**	
DHEA-S	0.003	0.975	0.034	0.749	−0.017	0.869	
FT	**0.339**	**0.001**	**0.227**	**0.030**	**−0.231**	**0.027**	
late postmenopausal women							
T	−0.055	0.680	0.119	0.373	−0.109	0.413	
SHBG	0.006	0.964	−0.064	0.631	0.077	0.564	
DHEA-S	**0.297**	**0.023**	**0.282**	**0.032**	**−0.277**	**0.035**	
FT	−0.050	0.710	0.128	0.340	−0.122	0.362	

Results are reported as Pearson’s rank value correlation coefficient (r-value).

Correlation is significant at the 0.05 level(P<0.05).

In the Pearson’s correlation analysis, SHBG levels correlated negatively with the T/L (r = −0.221, *P* = 0.034; Table 3) and the proportion of android fat (r = −0.279, *P* = 0.007; Table 3), whereas they correlated positively with the proportion of gynoid fat (r = 0.270, *P* = 0.009; Table 3) in early postmenopausal women after adjustment for age and BMI. The FT correlated positively with the T/L (r = 0.339, *P* = 0.001; Table 3) and the proportion of android fat (r = 0.227, *P* = 0.03; Table 3), while FT correlated negatively with the proportion of gynoid fat (r = −0.231, *P* = 0.027; Table 3) in early postmenopausal women. In the late postmenopausal group, after adjustment for age and BMI, the DHEA-S levels correlated positively with T/L (r = 0.297, *P* = 0.023; Table 3) and the proportion of android fat (r = 0.282, *P* = 0.032; Table 3), whereas they correlated negatively with the proportion of gynoid fat (r = −0.277, *P* = 0.035; Table 3).

The results from multiple linear regression analyses are shown in Table 4. In early postmenopausal women SHBG levels correlated negatively with the T/L (model^1^,β = −0.235, *P* = 0.023; Table 4) and the proportion of android fat (model^1^,β = −0.267, *P* = 0.007; Table 4), whereas they correlated positively with the proportion of gynoid fat (model^1^,β = 0.280, *P* = 0.006; Table 4).A higher FT in early postmenopausal women was significantly associated with a higher T/L (model^1a^,β = 0.367,*P* = 0.003;model^2^,β = 0.302,*P* = 0.002;model^2a^,β = 0.279,*P* = 0.005;Table 4),higher proportion of android fat(model^1a^, β = 0.283, *P* = 0.047;model^2^, β = 0.225, *P* = 0.030;model^2a^,β = 0.208,*P* = 0.043;Table4) and lower proportion of gynoid fat(model^1a^,β = −0.296,*P* = 0.047;model^2^,β = −0.244,*P* = 0.023;model^2a^,β = −0.223,*P* = 0.030;Table 4).Higher DHEA-S levels in late postmenopausal women were significantly associated with higher T/L (model^1,1a,2,2a^,β = 0.288, *P* = 0.026; Table 4),higher proportion of android fat (model^1,1a,2,2a^, β = 0.251, *P* = 0.047; Table 4) and lower proportion of gynoid fat (model^1,1a,2,2a^,β = −0.302, *P* = 0.019; Table 4).

**Table 4:** Associations of androgens with body fat distribution by multiple linear regressions analysis in early and late postmenopausal groups.

	T/L		Proportion of android fat		Proportion of gynoid fat		
	β-value	p-value	β-value	p-value	β-value	p-value	
early postmenopausal women							
Model^ 1^ SHBG	**−0.235**	**0.023**	**−0.267**	**0.007**	**0.280**	**0.006**	
Model ^1a^ FT	**0.367**	**0.003**	**0.283**	**0.047**	**−0.296**	**0.047**	
Model^ 2^ FT	**0.302**	**0.002**	**0.225**	**0.030**	**−0.244**	**0.023**	
Model^ 2a^ FT	**0.279**	**0.005**	**0.208**	**0.043**	**−0.223**	**0.030**	
late postmenopausal women							
Model^1,1a,2,2a^ DHEA-S	**0.288**	**0.026**	**0.251**	**0.047**	**−0.302**	**0.019**	

Results are reported as standardized regression coefficient (β). The Model^1^ used in analysis included T, DHEA-S, SHBG, BMI and age. The Model^1a^ used in analysis included T, FT, DHEA-S, SHBG, BMI and age. The Model^2^ used in analysis included DHEA-S, FT, BMI and age. The Model^2a^ used in analysis included DHEA-S, SHBG, FT, BMI and age.BMI and age are not shown in the table. Regression coefficient is significant at the 0.05 level (P<0.05).

## Discussion

Body fat is harmful to health depending on its distribution. There is a marked sex difference in the body fat distribution between men and women. Men tend to accumulate fat in the abdomen (android fat distribution), whereas women tend to accumulate fat in the gluteal-femoral region (gynoid fat distribution) [29].An increased android fat distribution and a decreased gynoid fat distribution which are thought to represent abdominal fat accumulation are risk factors for CHD and MS in women [30].

Natural menopause is associated with increased abdominal fat that exceeds changes normally attributed to the aging process, primarily in the perimenopausal year [8], [31], [32]. Evidences from estrogen therapy have demonstrated that the declined levels of estrogens after menopause are associated with abdominal fat accumulation [9].However, to date the relationship between endogenous androgens and body fat distribution is unclear.

Ovarian production of testosterone remains relatively stable in early postmenopause, in fact, the relative contribution of the ovarian testosterone to total testosterone increases [33]. SHBG is the main transport protein for testosterone to target tissues and modulates their biological activity. In this study, overweight and obese women of early postmenopausal group had lower SHBG levels than women with normal weight, which suggested that SHBG levels correlated negatively with abdominal fat accumulation in early postmenopausal women. Results from partial correlation and multiple linear regression analyses (in model^1^)confirmed this. Our results were in agreement with previous reports that low SHBG levels correlated with abdominal obesity in postmenopausal women [26], [34].

In our study, the FT correlated positively with abdominal fat accumulation in early postmenopausal women in model^2^. We further tested in model^1a,2a^ including FT and SHBG the relationship between androgens and body fat distribution. Interestingly, the FT was positively and significantly associated with abdominal fat accumulation though the strength of the association was a little different in different models, whereas the relationship between SHBG and body fat distribution was no longer significant. These results suggested that SHBG was not an independent factor associated with abdominal fat accumulation and the FT was independent from SHBG and T to predict abdominal fat accumulation.

In late postmenopause, the ovarian production of androstenedione declines, the DHEA-S from adrenal gland becomes the primary source of this precursor [35]. Serum DHEA-S levels decrease markedly during aging [15]. We also found that late postmenopausal women had lower DHEA-S levels than early postmenopausal women. Some researchers have reported that DHEA therapy in postmenopausal women could improve syndromes that result from androgen deficiency [36], [37]. DHEA is even sold over the counter in the USA as a nutritional supplement. However, due to the lack of definitive evidence for biological mechanisms and safety, DHEA therapy for postmenopausal is not recommended [38], [39].

The reduction of DHEA-S during aging results in a dramatic reduction in the formation of androgens in peripheral tissues, which is thought to be associated with obesity in men [40]. Conversely,this study showed that overweight and obese women had higher DHEA-S levels than normal weight women in the late postmenopausal group. The partial correlation and multiple linear regression analyses (in model^1,1a,2,2a^) further showed a positive relationship between DHEA-S and abdominal fat accumulation. Our findings were consistent with the findings that endogenous DHEA-S levels were positively associated with insulin resistance which is one of the complications of abdominal obesity [41]. This study suggested that DHEA therapy should be administered cautiously in late postmenopausal women, especially in women with overweight and obesity. Due to relatively higher endogenous DHEA-S levels in late postmenopausal women with overweight and obesity, exogenous DHEA therapy could cause more DHEA accumulation in peripheral tissues. As DHEA-S converts more readily to androgens than to estrogens [42], higher DHEA-S levels result in an increase in testosterone production in peripheral tissues, which may cause side effects(e.g. insulin resistance, acne, hypertrichiasis) due to higher testosterone.

We did not find a direct relationship between serum T levels and body fat distribution. This may be because the majority of circulating testosterone bounds to SHBG, and only FT expresses a biological effect. Serum T levels, therefore, do not fully represent the levels of bioavailable testosterone. Additionally, testosterone is mainly locally synthesized in the peripheral tissues from DHEA-S in late postmenopausal women without relying on transport by SHBG. After inducing their biological effects, most testosterone is inactivated immediately in the same cells [15], [43]. The serum levels of testosterone cannot indicate the levels of this bioavailable testosterone in peripheral tissues.

The proportion of body fat increases with aging and postmenopausal status is associated with abdominal fat accumulation [2], [44]. We also found that late postmenopausal women had a 1.81% higher proportion of body fat than early postmenopausal women due to aging. Body fat tended to accumulate in the abdomen in overweight and obese postmenopausal women. Previous studies indicated that the onset time of this tendency was early postmenopause in which the ovaries were hormonally active for secreting testosterone [2], [8]. Adipose tissue is known to express several steroidogenic and steroid-inactivating enzymes which covert DHEA-S to testosterone [45]. With ageing more testosterone is produced from DHEA-S and inactivated in adipose tissues rather than transported by SHBG from ovaries. Maybe the different sources of androgens accounted for the different correlations in early and late postmenopausal women. However, whether menopause-related changes in endogenous androgens account for body fat distribution or whether body fat distribution drives changes in endogenous androgens required further study.

In conclusion, our study suggested that the FT in early postmenopausal women and DHEA-S levels in late postmenopausal women correlated positively with abdominal fat accumulation.
