# Correction: The Relationship between Endogenous Androgens and Body Fat Distribution in Early and Late Postmenopausal Women

**DOI:** 10.1371/annotation/0ccac188-950f-4908-b232-35fb44ba7847

**Published:** 2013-10-16

**Authors:** Yuankui Cao, Shaofen Zhang, Shien Zou, Xian Xia

In Table 2, under the column heading "Late Postmenopausal Women," the first category title above "n = 53" should read "BMI < 24". 

